# Dr. Kenneth W. Millican

**Published:** 1904-10

**Authors:** 


### Dr. Kenneth W. Millican.

THE *New York Medical Journal* has enjoyed the rare fortune since its foundation many, many years ago of having for its editor-in-chief one of the most accomplished and erudite members of the medical profession, who may be regarded as the dean of the medical journalistic corps.

Dr. Frank P. Foster is everywhere recognised as embodying, as nearly as possible in human nature, the idealistic in medical editorship. During the past few years he has enjoyed the coadjutorship of Dr. Kenneth W. Millican as associate editor to whom, in no small degree, is due the recent advancement of the journal. It had outgrown the editorial management of any one man, no matter how able, and Dr. Foster was fortunate in summoning to his aid a man of such rare mental and physical equipment as Dr. Millican possesses. During Dr. Foster's long illness last winter and spring the editorial affairs of the journal were conducted with such rare skill and judgment that but few, we feel justified in saying, knew that the senior editor temporarily had taken his hand off the helm.

The foregoing is but a prologue,—a curtain raiser, in the language of the day,—to the real purpose of this writing. Dr. Millican's abilities, as might have been expected, attracted the attention of other journals and publishers, and so, in the course of time he was invited to other fields. Among his flattering offers came one from the *Saint Louis Medical Review,* a weekly of ancient lineage and honorable career. The terms of this proposal were so liberal that Dr. Millican felt they were not to be declined; and so he has betaken himself to the city of the Louisiana Purchase Exposition to become installed as editor of the *Review,* with its first issue in this month of October.

Dr. Millican's talents will have opportunity in this new field to develop the periodical of which he assumes control, into an influential journal that shall represent the medical profession in the west and south; indeed, its field should begin with the Ohio and Mississippi valleys and reach to the Pacific slope. We congratulate the *Review* upon the career that awaits it under its new editorial management, and extend to Dr. Millican our cordial good wishes in his new undertaking.

But, alas for Dr. Foster! We fear he will not easily find so able and useful an associate.

---

WHAT appears to be an authentic case of syphilitic reinfection occurring six years after previous manifestation of a syphilitic attack is reported by Klotz. The symptoms yielded to mixed treatment, as reported by the *Journal of the American Medical Association,* with recurrent exacerbations in the form of gummatous swellings over the ribs, bones of the leg, finger, and the like. In the initial attack there was no history of chancre, although in the reinfection this was quite evident.

Klotz sees no reason to doubt the authenticity of the case, there being a typic primary lesion, followed by glandular enlargement, a rash, iritis, a papular eruption and finally hemiplegia.

If, as Klotz believes, the final attack was true syphilis, the case gives good ground for his belief that tertiary manifestation of syphilis may be directly produced through inoculation from a tertiary lesion in another individual.

---

THE article on Chinese Medicine in this issue was originally written by Mrs. Bishop for publication in the *Anglo-Saxon Review.* It is one of the most comprehensive articles on the subject in brief form which has yet appeared.

The added notes on Chinese medicine are based on the personal experiences of the author during several years residence among the Chinese in New York. That portion referring to Chinese Surgery is properly credited.

---

FEW people know that in Paris there is very little garbage collected, for the very good reason that there is no waste. The surplus and remnants of food from the tables of cafés, hotels, and even private families, are used in a state just below themselves, that the remnants of these second tables, let us call them, supply a third grade and so on down to the poorest hovels, until all the nourishment is stewed, boiled and scraped from every morsel of food. The system is almost absolutely perfect.

The sale of certain proprietary medicines has been enormous and inexplicable save through their extraordinary testimonials, some of which must have puzzled even physicians whose attention may have been drawn to them. An explanation is now forthcoming through an associated press communication, says an editorial in the *New York Medical Journal,* which we transcribe:

Remarkable testimony has been obtained by the postoffice department as to the ways in which testimonials are obtained by some of the big concerns engaged in this business. One large firm admitted that it had agents out seeking persons who had formerly occupied prominent positions in the community, but had suffered financial reverses and were harassed by debts they were unable to settle. The agents would obtain possession of the unpaid accounts, and would then apply pressure to the unfortunate victims, demanding immediate payment in full. Finally, after long persecution, the desperate victim would be invited or commanded to call at the office of an attorney, where he would be given to understand that if he would sign and swear to a testimonial a receipt in full for the claims against him would be given. This seems incredible, but the facts are now on file in the records of the postoffice.

It is hard to believe in such villainy. It is abominable that the article from which we make the foregoing quotation is headed in the lay press, "Methods of Medical Men."

---

Two very sensible suggestions are made by Kossman in the *Centralblatt für Gynekologie* in discussing the liability of physicians and surgeons to attack on the part of blackmailers and shyster lawyers through malpractice suits. He advocates the signing of a paper placing the patient in the surgeon's hands, to do as his best judgment dictates. When a surgeon hears of the contemplation of a suit for damages, he should bring suit for libel, thus placing the prospective plaintiff on the defensive, and invariably turning the tables.

---

The Institute of Medical Research, endowed by John D. Rockefeller, is erecting a new laboratory in New York City, which will be five stories high, 136 feet wide and 60 feet deep, with a facade of limestone and brick, and having a porch entrance flanked with decorative columns for the support of electric lights. The first floor will contain an assembly hall, a library and study, and the directors' room. The upper floors will contain a series of large general and special laboratories and research rooms. The top floor will have a dining hall and living quarters, and the roof will have a special operating room and quarters for the animals under examination. A two-story building for animals and for electric power will adjoin the main structure. The cost will be over $300,000.

---

ELSEWHERE in this issue—namely, under "Topics of Public Interest," we publish three articles from the *New York State Journal* of Medicine for September, 1904. The first two are explanatory of the status of the unification scheme and show why it was not accomplished as well as indicate what is still to be done to bring about amalgamation between the two state medical organisations. The third is an editorial commenting upon the situation. We confess that, just at this delicate juncture, we should not have written the latter had we been in the editor's place, but we gladly publish it that our readers may be advised of the different viewpoints concerning unification in the state of New York, in order to be able to judge accurately of the motives that are employed either to promote or retard it.

---

THE statue to be erected to the memory of Dr. William E. B. Davis, late of Birmingham, Ala., to which we have before referred in these columns, is nearly completed. It will be placed in Capitol Park, in the city of Birmingham, during this autumn, and will be unveiled December 16, 1904, during the meeting of the Southern Surgical and Gynecological Association in that city.

The model has already been accepted by the artist, G. Moetti, the noted sculptor of New York, and has received the approval of Mrs. Davis and other relatives. It has been cast in bronze and will stand twenty feet high, measuring from the base of the pedestal. It will become an enduring monument to the famous young surgeon, who left behind an imprint on the guild of medicine more enduring than granite, brass, or bronze.

